# Risk factors of multi-drug resistant organism in patients hospitalized with community acquired pneumonia at Wahidin Sudirohusodo Hospital

**DOI:** 10.1017/ash.2025.141

**Published:** 2025-09-03

**Authors:** Suriana Dwi Sartika, Sudirman Katu, Risna Halim

**Affiliations:** Division of Tropical Infections, Department of Internal Medicine, Faculty of MedicineHasanuddin University

**Keywords:** risk factors, multidrug resistant organism, community acquired pneumonia

## Abstract

**Objectives:** Infection with antibiotic resistant organisms has become a global problem, including in cases of pneumonia. Multi drug resistance organism (MDRO) has an impact on mortality, morbidity, and health costs. There are several risk factors that play a role in the incidence of MDRO in community acquired pneumonia. The purpose of this study was to analyze the risk factors for the incidence of MDRO in hospitalized patients with community acquired pneumonia at Wahidin Sudirohusodo Hospital. **Methods:** This study used an analytic observational method with a retrospective cohort. Data were taken from patient medical records from July-December 2023. **Results:** There were 49 (46.7%) MDRO and 56 (53.3%) non-MDRO. Based on statistical tests, MDRO infection is associated with comorbid malignancy (p value 0.002) and cardiovascular comorbidities (p value 0.015). The most common pathogens found were Acitenobacter baumanii (22.8%) and Klebsiella pneumonia (20%). **Conclusions:** Risk factors associated with the incidence of MDRO in community acquired pneumonia patients are malignancy and cardiovascular disease.

